# A Systematic Review on Vaccine Hesitancy in Black Communities in Canada: Critical Issues and Research Failures

**DOI:** 10.3390/vaccines10111937

**Published:** 2022-11-15

**Authors:** Jude Mary Cénat, Pari-Gole Noorishad, Schwab Mulopo Bakombo, Olivia Onesi, Aya Mesbahi, Wina Paul Darius, Lisa Caulley, Sanni Yaya, Marie-Hélène Chomienne, Josephine Etowa, Vivek Venkatesh, Rose Darly Dalexis, Roland Pongou, Patrick R. Labelle

**Affiliations:** 1 School of Psychology, University of Ottawa, Ottawa, ON K1N 6N5, Canada; 2 Interdisciplinary Centre for Black Health, University of Ottawa, Ottawa, ON K1N 6N5, Canada; 3 Faculty of Social Sciences, University of Ottawa, Ottawa, ON K1N 6N5, Canada; 4 Interdisciplinary School of Health Sciences, University of Ottawa, Ottawa, ON K1N 6N5, Canada; 5 Faculty of Medicine, Family Medicine University of Ottawa, Ottawa, ON K1N 6N5, Canada; 6 School of International Development and Global Studies, Ottawa, ON K1N 6N5, Canada; 7 The George Institute for Global Health, Imperial College London, London NW9 7PA, UK; 8 Institut du Savoir Montfort, Ottawa, ON K1K 0T2, Canada; 9 School of Nursing, University of Ottawa, Ottawa, ON K1N 6N5, Canada; 10 Department of Art Education, Concordia University, Montreal, QC H3H 1M8, Canada; 11 Department of Economics, University of Ottawa, Ottawa, ON K1N 6N5, Canada; 12 Library, University of Ottawa, Ottawa, ON K1N 6N5, Canada

**Keywords:** vaccine hesitancy, racial disparities, Black communities, Canada, systematic review

## Abstract

Black communities have been disproportionately impacted by Coronavirus Disease 2019 (COVID-19) in Canada, in terms of both number of infections and mortality rates. Yet, according to early studies, vaccine hesitancy appears to be higher in Black communities. The purpose of this systematic review is to examine the prevalence and factors associated with vaccine hesitancy in Black communities in Canada. Peer-reviewed studies published from 11 March 2020 to 26 July 2022, were searched through eleven databases: APA PsycInfo (Ovid), Cairn.info, Canadian Business & Current Affairs (ProQuest), CPI.Q (Gale OneFile), Cochrane CENTRAL (Ovid), Embase (Ovid), Érudit, Global Health (EBSCOhost), MEDLINE (Ovid), and Web of Science (Clarivate). Eligible studies were published in French or English and had empirical data on the prevalence or factors associated with vaccine hesitancy in samples or subsamples of Black people. Only five studies contained empirical data on vaccine hesitancy in Black individuals and were eligible for inclusion in this systematic review. Black individuals represented 1.18% (*n* = 247) of all included study samples (*n* = 20,919). Two of the five studies found that Black individuals were more hesitant to be vaccinated against COVID-19 compared to White individuals, whereas the other three found no significant differences. The studies failed to provide any evidence of factors associated with vaccine hesitancy in Black communities. Despite national concerns about vaccine hesitancy in Black communities, a color-blind approach is still predominant in Canadian health research. Of about 40 studies containing empirical data on vaccine hesitancy in Canada, only five contained data on Black communities. None analyzed factors associated with vaccine hesitancy in Black communities. Policies and strategies to strengthen health research in Black communities and eliminate the color-blind approach are discussed.

## 1. Introduction

Referred to as the “great equalizer” at its beginning, the coronavirus disease 2019 (COVID-19) pandemic has highlighted and exacerbated existing racial disparities in the health care systems of different provinces in Canada [1,2,3,4]. People from Black communities have been particularly affected by these disparities, in terms of the number of both infections and deaths [1,2,3,5,6]. A report from Statistics Canada revealed that mortality rates related to COVID-19 were higher in Black communities than in other communities (49 deaths per 100,000 population for Black people vs. 22 deaths per 100,000 for White population) and were 2.2 times higher among Black people compared to White and non-Indigenous people in Canada [7]. In addition, reports from various public health agencies in the province of Ontario, which has more than 50% of Canada’s Black population, indicate an overrepresentation of COVID-19 cases in Black communities [8,9]. Data from the City of Toronto showed a continuous overrepresentation in the number of COVID -19 cases in the Black population since June 2020, when Toronto started collecting racial data. The trend persisted each month of the pandemic in Toronto, with the number of cases, reaching 33% in August 2020, even though the Black population represents only 9% of the city’s population [9]. In Ottawa, the second largest city of the province, similar observations were made as Black people constituted 37% of cases while representing only 7% of the population [8]. Studies conducted in other cities, such as Montreal and Edmonton, also corroborated that people from Black communities were more impacted by the COVID-19 pandemic [10,11].

Triggered by various major events (e.g., the deaths of George Floyd and Joyce Echaquan, the Black Lives Matter movement), the COVID-19 pandemic has prompted a series of calls to take into account the structural inequalities in society while reporting on health disparity research in Canada [12,13,14]. Although access to health care is guaranteed by legislative provisions in all Canadian provincial and territorial health care systems, Black communities continue to face disparities in public health issues and in the care they receive in both physical and mental health settings [14,15,16,17]. Indeed, individuals from Black communities face particular disparities in hypertension, diabetes, cancer care, depression, anxiety, post-traumatic stress disorder, and are less likely to have a family physician [15,17,18,19,20,21,22,23]. One important issue that has been raised during the pandemic crisis is that of vaccination rates among Black communities [24,25]. Indeed, a study conducted by Statistics Canada revealed that people from Black communities are the least likely to be vaccinated (56.4%) compared to White (77.7%) and South Asian (82.5%) people [24]. Despite free distribution of the vaccine by the Canadian federal government to all provinces and territories, which have adopted various strategies to make the vaccine available (e.g., mobilization of gymnasiums, sports centers, convention centers, neighborhood pharmacies, and schools for vaccination), many people in Black communities remained reluctant to be vaccinated [24,25]. In response to these initial observations, several initiatives have been undertaken by different public health agencies and organizations to encourage vaccination in Black communities [25,26,27]. However, how have health researchers in Canada tried to understand the factors associated with vaccine hesitancy among Black communities? What evidence is available on factors associated with vaccine hesitancy in Black communities? These questions are even more relevant in the Canadian context given that in the neighboring country, the United States, a systematic review showed that Black people were 3.14 times more likely to refuse to be vaccinated compared to White people [28]. Another systematic review found that vaccine hesitancy was 41.6% among African Americans compared to 26.3% in the general population [29]. To review the state of the Canadian health research on these issues, we conducted a systematic review of peer-reviewed studies to examine the prevalence and factors associated with vaccine hesitancy in Black communities in Canada.

## 2. Methods

### 2.1. Protocol and Registration

This systematic review has been registered with PROSPERO (#CRD42022320695), with no other similar reviews registered to date. This review was reported in accordance with the 2020 Preferred Reporting Items for Systematic Reviews and Meta-analyses (PRISMA) reporting guideline [30].

### 2.2. Search Strategy and Information Sources

Studies reporting prevalence and factors associated with COVID-19 vaccine hesitancy among Black Canadians were identified. In consultation with the research team, a research librarian (PRL) developed a search strategy to find relevant published articles through eleven electronic databases: APA PsycInfo (Ovid), Cairn.info, Canadian Business & Current Affairs (ProQuest), CPI.Q (Gale OneFile), Cochrane CENTRAL (Ovid), Embase (Ovid), Érudit, Global Health (EBSCOhost), MEDLINE (Ovid), and Web of Science (Clarivate).

The search strategies of previous reviews on COVID-19 [31,32,33] and on vaccine hesitancy [34,35,36], as well as other COVID-19 search strategies from the Medical Library Association [37] were consulted and adapted for this systematic review. Search lines relating to Canada and its provinces and territories were also included. The final search strategy combined relevant subject headings and keywords, and results were limited while searching for those published since 2020. The strategy for MEDLINE (Ovid) was peer-reviewed by another research librarian in concordance with the Peer-Review of Electronic Search Strategy guideline [38]. The final strategy was executed on July 26, 2022. In addition to the database searches, a streamlined search strategy was executed on LitCovid, an up-to-date curated list of references related to research on COVID-19. More detailed information on the implemented search strategy can be found in Supplementary File S1. After running the searches, citations were imported into Covidence, an online tool used to manage various steps of a systematic review’s screening phases.

### 2.3. Selection Criteria

We included studies that met the following inclusion criteria: (1) reported the nature, incidence, prevalence (including intention to be vaccinated), risk and protective factors, and disparities associated with vaccine hesitancy among Black people; (2) published in English or French; (3) peer reviewed empirical studies (quantitative, qualitative, mixed methods); and (4) conducted in Canada.

### 2.4. Steps for Selection

**Figure 1:**
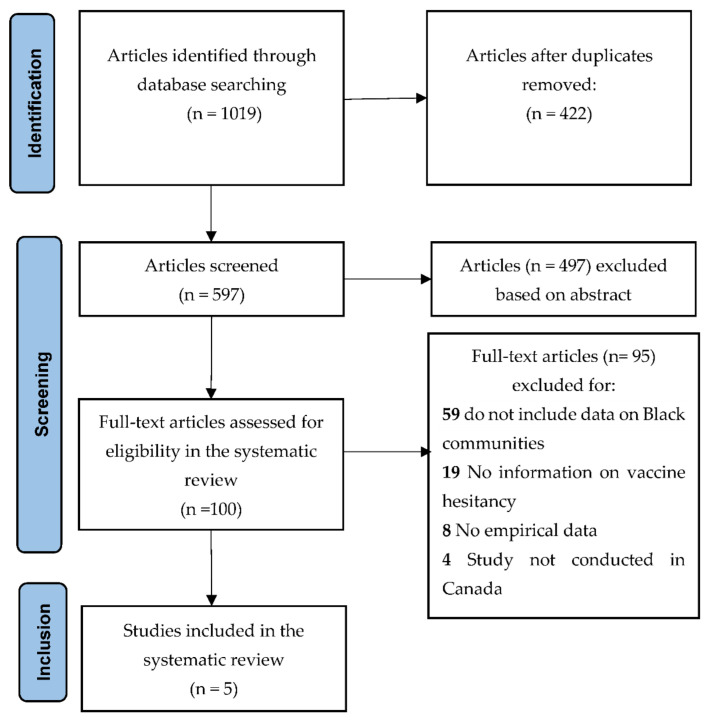
PRISMA flow diagram of COVID-19 vaccine hesitancy in Black communities in Canada.

The title and abstract of publications and their full text were independently evaluated by two authors for inclusion (OO, AM). Two authors (JMC, PGN) resolved discrepancies in the screening of the abstracts and full texts. Figure 1 presents the PRISMA flow diagram.

### 2.5. Quality Assessment

The methodological quality of studies, including bias, conduct, and analysis, was assessed using the JBI Critical appraisal checklists for Qualitative and Cross-sectional Research [39,40]. Eight criteria, including appropriateness of the sample frame, recruitment procedure, adequacy of the sample size, description of participants and setting were evaluated for quantitative papers: (1) Were the criteria for inclusion in the sample clearly defined? (2) Were the study subjects and the setting described in detail? (3) Was the exposure measured in a valid and reliable way? (4) Were objective, standard criteria used for measurement of the condition? (5) Were confounding factors identified? (6) Were strategies to deal with confounding factors stated? (7) Were the outcomes measured in a valid and reliable way? (8) Was appropriate statistical analysis used? A single point was attributed to studies for each criterion met. We included studies with 5 or more points. Two authors (OO, AM) independently assessed the quality of the articles and one author (PGN) validated (see Table 1).

### 2.6. Data Extraction

Study details pertaining to the year of publication, methodology, sample characteristics, description of measures used, main findings, and quality of assessment were extracted independently by two (OO, AM), then verified and validated by two other authors (PGN, SMM) and the principal investigator (JMC). Data extraction was performed in Microsoft Office Excel using a standardized data extraction form (See Table 1).

## 3. Results

### 3.1. Characteristics of Included Studies

The PRISMA flow diagram of study retrieval and selection is provided in Figure 1. A total of 1019 studies were imported into Covidence for screening. After removing duplicates (*n* = 422), two authors screened the title and abstracts of the 597 studies, excluding 497 of them. One hundred full texts were assessed for eligibility in the systematic review and 95 were excluded (more details are provided in Figure 1). A total of five cross-sectional studies underwent qualitative reviews for the current study [41,42,43,44,45]. The characteristics and main findings of these studies are presented in Table 1. All participants in the included studies were 20,919 individuals living in Canada, with 247 (1.18%) identifying as Black (Black communities in Canada represent 3.5% of the national population).

### 3.2. Narrative Synthesis

Among the five studies included in this systematic review, two of the studies reported that Black people were significantly more hesitant to receive a COVID-19 vaccine compared to White people in Canada [41,42], whereas three studies reported no significant racial differences in vaccine hesitancy [43,44,45].

In one of the studies on vaccine hesitancy among racial communities in Canada, New York, California, Texas, and Florida, a single-item questionnaire asked how likely participants were to get vaccinated if a vaccine for COVID-19 became available (answer options ranged from “1, Definitely” to “6, Definitely Not”) [41]. The mean scores differed by race among Canadians for this questionnaire (Indigenous = 3.1, Black = 3.4, Latinx = 2.6, East Asian = 2.5, White = 2.2). In Canada, vaccine hesitancy was higher among Black and Indigenous people compared to White people (F(4, 1679) = 11.63, *p* < 0.001), but no significant differences were found between Latinx and White people. Similar results were observed for vaccine mistrust which was higher among Black (3.1) and Indigenous (3.0) people, compared to White individuals (2.3), F(4,1679) = 9.38, *p* < 0.001. Kaida and colleagues also found higher rates of vaccine hesitancy among African/Caribbean/Black (42.9%), Indigenous (34.9%), and multiracial people (22.2%) compared to White people (19.1%; reference group, *p* < 0.0001) among women living with HIV in British Columbia, Canada [42].

Lunsky and colleagues examined beliefs regarding COVID-19 vaccines among Canadian workers in the intellectual disability sector prior to vaccine implementation [43]. In the full sample, more than half of respondents (62%) reported that they were “very likely” and 20% reported that they were “somewhat likely” to get a COVID-19 vaccine. Concerning race, 9.4% of African or Caribbean participants, 0.9% of Indigenous, First Nations, or Metis participants, 3.0% of Latin participants, 14.3% of multiracial participants, and 10.6% of European participants endorsed being “very unlikely” to receive a COVID-19 vaccine. No significant race differences were found. A study also found no racial differences in vaccine hesitancy among Black communities in Canada [44]. Among their sample of Canadians, 67.7% of Black participants were willing to receive the vaccine compared to 79.9% of non-Black participants [44]. The likelihood to receive a COVID-19 vaccine was lower among Black individuals (OR = 0.53) but was not significant (*p* = 0.13).

Moreover, another study found no significant race differences concerning vaccine hesitancy among public-school teachers in British Columbia, Canada [45]. Most public-school teachers (89.7%) reported they were likely or very likely to accept a COVID-19 vaccine. Among 25 Black participants, 76% (*n* = 19) intended to receive a COVID-19 vaccine.

Results from the included studies do not permit us to draw comparisons based on intersections of race and other sociodemographic characteristics, including gender, age, education level, socioeconomic status, income, and employment status.

## 4. Discussion

The purpose of this article was to examine the prevalence and factors associated with vaccine hesitancy in Black communities in Canada. Of the five studies that were included in this systematic review, two found that individuals from Black communities were more likely to be vaccine hesitant compared to White individuals [41,42], while the other three found no significant differences between racial groups [43,44,45]. Even in studies where there were no significant differences, the proportion of vaccine hesitancy is higher among Black people. However, these studies may be underpowered to detect the desired difference due to a smaller sample size. In addition, Black individuals represented only 1.18% of the sample of the five included studies.

Although the included studies demonstrated a tendency for greater hesitancy to receive the COVID-19 vaccine among Black individuals, the current literature does not provide quantitative evidence of the prevalence or contributing factors to vaccine hesitancy in Black communities in Canada. Another systematic review examining vaccine hesitancy related to COVID-19 in Canada was able to include 29 studies conducted since the beginning of the pandemic [46]. However, only five studies incorporated analyses that included Black communities among all the studies conducted on the important issue of vaccine hesitancy. Moreover, of the five included studies, three were conducted in British Columbia, where only 3.6% of the Black population resides [42,44,45], one was conducted in Ontario [43], and one included a subsample from English-speaking Canada [41]. None of the studies have identified French-speaking Black people, and in Gerresten’s study, Quebec, which is where more than a quarter of the Black population in Canada lives, was not included. In addition, all the studies were conducted before the COVID-19 vaccine implementation; none analyzed the situation of Black people during the COVID-19 vaccination campaign in Canada. In the same way, none of these studies has addressed the critical situation of Black children and adolescents in regard to vaccine hesitancy. Similarly, we found that no studies have been conducted exclusively among Black communities to examine in detail the factors associated with vaccine hesitancy.

In view of the small sample sizes of the Black population (varying from 21–125), the included studies were also unable to examine racial issues according to sociodemographic data (e.g., gender, age, education, income) and other factors that may be associated with vaccine hesitancy. Such studies are important, knowing that Black individuals tend to have a lower socioeconomic status compared to White communities and that socioeconomic status is an important factor in vaccine hesitancy, but also in case and mortality rates related to COVID-19 in Canada [7]. A systematic review of vaccine hesitancy among African Americans and Hispanics in the US found that major predictors of vaccine hesitancy were gender, age, income, education, household size, medical distrust, experiences of racial discrimination, exposure to COVID-19 myths and misinformation, concerns about side effects, and vaccine safety issues, among others [29]. Similarly, studies should also examine the impact of the organization of the vaccination campaign in different provinces in Canada and its effects on vaccination coverage in Black populations. It will be especially important to know the impact of the preferred means of booking appointments via internet platforms on vaccination coverage in different ethnic groups and analyze other factors such as immigrant status, stigmatization, and mental health [47,48,49].

### 4.1. Strengths and Limitations

One of the most important strengths of this review is to highlight that, even in 2022, health research in Canada continues to use a color-blind approach that does not consider racial issues and does not examine nor collect data accordingly, nor does it ensure sufficient or oversampling of Black communities. Another strength resides in the inclusion of studies that have undergone rigorous peer review. However, this introduces an important limitation as the inclusion of grey literature could have allowed for the analysis of a wider range of results and data from public health agencies as well as from Health Canada and Statistics Canada.

### 4.2. Implications for Policy, Interventions, and Research

This systematic review could not find sufficient evidence on the prevalence and factors associated with vaccine hesitancy among Black Canadian communities. However, it offers different avenues for health research, research funding agencies, and scientific journals in Canada to improve our knowledge of Black communities living in Canada. We suggest that research funding agencies:

1. Require a clear plan from researchers on how they will disaggregate their data by race and analyze racial issues and related sociodemographic characteristics in their research proposal, in the same way that many funding agencies presently require data analysis plans based on gender and sex.
2. Require researchers to make efforts to recruit Black people into their studies in ways that are scientifically sound with sufficient sampling and power (including Francophone and Anglophone Black individuals); and to be inclusive, collaborative, culturally appropriate, humble, and respectful of Black communities.
3. Require researchers to address data analysis and racial issues in the end-of-research report. Researchers who have failed to analyze racial issues and have not provided evidence of having made the necessary efforts to do so may be limited for a certain period of time before they can submit new funding proposals or must have as a co-principal investigator who has been able to conduct analyses in accordance with racial issues with their team.
4. Require principal investigators to include co-researchers from Black communities in their projects. However, measures must be taken to ensure that researchers do not do this as a check box as it is often currently done. Researchers must report in their final report on efforts to include Black co-investigators in all stages of the research, including publication.

Scientific, publishers, journals and editors should:

1. Develop racial diversity and inclusion requirements for authors.
2. Develop clear and stringent policy for requiring the analysis of racial data for each article submitted. Authors who have not included race-based data and analyses should add a note in their manuscript (to be published) stating why their analyses were not disaggregated by race or why the study did not consider racial issues or why it was not relevant for their article.

### 4.3. Conclusions

These measures and others should avoid the status quo where health research in Canada continues to use a color-blind approach. It will help to embrace an analytical framework that captures the complexity of racial issues [50]. Although the present review indicates a tendency toward greater reluctance to be vaccinated against COVID-19 in Black communities, it fails to provide evidence on associated prevalence and factors. However, while there is a sense that the critical period of the COVID-19 crisis is behind us, the need for studies on vaccine hesitancy in Black communities remains, to address COVD-19 as well as future epidemics. Many people from Black communities who previously had no hesitancy to vaccinate their children according to physician and public health recommendations have now become skeptical of all vaccines [51]. Future research that includes Black communities and analyzes racial issues in detail is needed to evaluate the damage that vaccine hesitancy related to COVID-19 will have on the immunization of Black communities against various infectious diseases and in the coming years in Canada and elsewhere.

## Figures and Tables

**Table 1:** Key Characteristics of Included Studies and Main Findings.

Authors (Year)	Sample (Female %)	Age	Race/Ethnicity (%)	Measures and Analyses	Main Findings	Quality Evaluation
Gerretsen et al. (2021) [41]	*Full sample* *n* = 4434 (50.38%) *Canadian subsample* *n* = 1936 *Black Canadian subsample* *n* = 48	*Full sample* Age range = 18–65+ M_age_ = 48.7 (*SD* = 17.2) *Black Canadian subsample* Age range = 18–65+ M_age_ = 36.7 (*SD* = 16.2)	*Full sample* 74.4% White 11.9% East Asian 7.6% Latinx 4.9% Black 1% Indigenous	Measures. Participants’ degree of vaccine hesitancy was assessed using a single item that asked how likely they are to get vaccinated if a vaccine for COVID-19 becomes available. The answer options ranged from “1, Definitely” to “6, Definitely Not,” with a higher score representing greater hesitancy. Sociodemographic questionnaire; Vaccine complacency; Vaccine confidence. Analyses. One-way Analysis of variance (ANOVA); Chi-Square tests and Multivariate analyses of variance (MANOVA).	*Full sample* Among the full sample (Canada and the United States), 43.7% of Indigenous, 33.4% of Black, and 56.5% of Latinx participants were “very probably” to “definitely” likely to get a COVID-19 vaccine compared to 59.6% of East Asian and 67.4% of White participants. Most people were supportive of the COVID vaccine. Education and political leaning influenced views on vaccines. *Black subsample* Self-identified Black respondents reported the most vaccine hesitancy compared to other races. The mean scores differed by race among Canadians for this questionnaire (Indigenous = 3.1, Black = 3.4, Latinx = 2.6, East Asian = 2.5, White = 2.2). In Canada, vaccine hesitancy was higher among Black and Indigenous people compared to White people (F(4, 1679) = 11.63, *p* < 0.001). Black (3.1) and Indigenous (3.0) individuals were more likely to declare “mistrust in vaccine benefit” compared to Latinx (2.9), East Asian (2.6) and White (2.3) groups, F(4, 1679) = 9.38, *p* < 0.001.	6/8
Kaida et al. (2022) [42]	*Full sample* *n* = 5588 (99.6%) BC residents *Black subsample* *n* = 21	*Full sample* Age range = 25–69 M_age_ = 48.2 (*SD* = 12.1)	79.5% White 13.9% Other/Mixed 3.3% Indigenous 0.04% Black	Measure. A modified WHO *Vaccine Hesitancy Scale* which included two factors: Lack of vaccine confidence and Vaccine risk. The primary outcome was intention to vaccinate (“If a COVID-19 vaccine were to become available to the public, and recommended for you, how likely are you to receive it?”), which was measured on a 5-point Likert scale. Attitudes toward the COVID-19 vaccine; Perceived behavioral control; Direct social norms; Indirect social norms. Analyses. Descriptive analyses (means, frequencies), Chi-square and fisher test; logistic regression.	*Full sample* The intention to be vaccinated was significantly lower in those living with HIV than those not living with HIV. This association was not statistically significant after adjusting for ethnicity, income, education, and essential worker status. 79.7% of participants reported being “very or somewhat likely” to receive a COVID-19 vaccine if it was to become available to the public and if it was recommended for them. *Black subsample* Intention to vaccinate was lower among racialized individuals compared to White people (African/Caribbean/Black = 42.9%, Indigenous = 34.9%, multiracial = 22.2%, White = 19.1%).	7/8
Lunsky et al. (2021) [43]	*Full sample* *n* = 3371 (84.7%) Canadian (Ontario) workers in the intellectual disability sector *Black subsample* *n* = 125 (4.9%)	*Full sample* Age range = 18–50+ 18–29 *n* = 604 30–39 *n* = 789 40–49 *n* = 763 50+ *n* = 1147	81.3% European 4.9% African or Caribbean 4.5% Asian 3.6% Indigenous, First Nations, or Metis 3.3% Unknown 1.3% Latin 1.1% Mixed	Measures. Vaccination intent was measured by the question “How likely is it that you would get a COVID-19 vaccine when it is offered?”, with four answer options: “very likely”, “somewhat likely”, “somewhat unlikely”, and “very unlikely”. Sociodemographic questionnaire; Information about vaccine beliefs; Who people consider to be the most trusted sources of COVID-19 information; Which media platforms people turn to for reliable COVID-19 information. Analyses. Descriptive analyses; univariate and multivariable logistic regression	More than half of respondents (62%) reported that they were ‘very likely’ to get a COVID-19 vaccine, and 20% reported that they were ‘somewhat likely’ to do so. The remainder were ‘somewhat unlikely’ (7%) or ‘very unlikely’ to get vaccinated (11%). There was no significant difference in vaccine intent between ethnicities in this study; 9.4% of African or Caribbean participants, 0.9% of Indigenous, First Nations, or Metis participants, 3.0% of Latinx participants, 14.3% of multiracial participants, and 10.6% of European participants endorsed being “very unlikely” to receive a COVID-19 vaccine.	6/8
Ogilvie et al. (2021) [44]	*Full sample* *n* = 4948 (84.8%) BC residents *Black subsample* *n* = 28	*Full sample* Age range = 25–69, M_age_ = 51.8 (*SD* = 10.5) 25–29 *n* = 111 (2.2%) 30–39 *n* = 573 (11.6%) 40–49 *n* = 1260 (25.5%) 50–59 *n* = 1496 (30.2%) 60–69 *n* = 1370 (27.7%) missing *n* = 138 (2.8%) 84.8% women	82.6% White 7.3% Asian 2.6% Indigenous 2.0% South Asian 1.3% Latin American 0.6% Black	Measures. 9-item Vaccine Hesitancy Scale (VHS), assessing lack of vaccine confidence and vaccine risk. Sociodemographic questionnaire; Vaccine attitudes; Direct Social norms; Indirect social norms; Perceived behavioural controls Analyses. Descriptive statistics; reliability analyses of the measure; mixed-effects logistic regressions.	*Full sample* Most adults, especially older individuals (>60 years), were more likely to receive a COVID-19 vaccine if available. In the full sample, 79.8% were ‘somewhat or very likely’ to receive a COVID-19 vaccine if it was available to the public and recommended for them. Those with less than high school education, along with those who report higher lack of confidence in vaccines and higher perceived risk of vaccines were less likely to indicate an intention to vaccinate. *Black subsample* Those who identified as non-White (AOR = 0.76) or Indigenous (AOR = 0.58) indicated that they are less likely to receive a COVID-19 vaccine. However, 67.7% of Black participants were willing to receive vaccine, compared to 79.9% of non-Black participants. The likelihood to receive a COVID-19 vaccine was not significantly different between Black (OR = 0.53, *p* 0.13) and non-Black participants (reference).	6/8
Racey et al. (2021) [45]	*Full sample* *n* = 5076 (74.9%) BC public School teachers *Black subsample* *n* = 25 (0.5%)	*Full sample* Age range = 20–60+ 20–30 *n* = 494 (9.7%) 30–40 *n* = 1326 (26.1%) 40–50 *n* = 1633 (32.2%) 50–60 *n* = 1234 (24.3%) 60+ *n* = 304 (6.0%) missing *n* = 85 (1.7%) 74.9% women	84% White 8.6% Asian 3.7% South Asian 2.9% Indigenous 0.7% Latin American 0.5% Black	Measures. 9-item Vaccine Hesitancy Scale (VHS), assessing lack of vaccine confidence and vaccine risk. Sociodemographic questionnaire; Ever delayed or refused an immunization; Sources of Information; COVID-19 is a serious illness; Vaccine knowledge. Analyses. Descriptive statistics; reliability analyses of the measure; Fisher’s and Kruskal-Wallis tests; logistic regressions.	Most reported they would be very likely to get a COVID-19 vaccine, with a vaccine hesitancy prevalence of only 5.71% for the full sample. Respondents reported seeking general vaccine information from reliable information sources: public health (78.1%), government websites (63.6%), and health care providers (57.3%). 40.7% of respondents reported professional organizations such as school boards or the teacher’s union as a source of vaccine information. Few respondents reported seeking general information about vaccines from social media. Respondents who were male and had an educational background in science or engineering and sought vaccine information from reliable information sources, including public health, school boards or teachers’ unions and health care providers were more likely to intend to receive a COVID-19 vaccine. Those who delayed a previous vaccine were less likely to intend to accept a COVID-19 vaccine. Age was not significantly related to vaccine hesitancy. There was no significant association between vaccine intention and visible minority status. Among 25 Black participants, 76% (*n* = 19), *p* = 0.04 intended to receive a COVID-19 vaccine.	7/8

## Supplementary Materials

The following supporting information can be downloaded at: https://www.mdpi.com/article/10.3390/vaccines10111937/s1, File S1: Search strategy.
