# Supplementary material for: A Systematic Review on Vaccine Hesitancy in Black Communities in Canada: Critical Issues and Research Failures
Source: Vaccines (Basel). 2022 Nov 15;10(11):1937. doi: 10.3390/vaccines10111937 (PMC9695687; doi:10.3390/vaccines10111937)
Supplement: Supplementary file 1 [file vaccines-10-01937-s001.zip › vaccines-1948094-supplementary.pdf]

List of Supplementary Files

1. Search strategy

Search strategy

The following databases were search on July 26, 2022:

- APA PsycInfo (Ovid, 1806 to July Week 4 2022) – 12 results
- Cairn – 3 results
- Canadian Business & Current Affairs (ProQuest, 1933 - current) – 74 results
- Canadian Periodicals Index (Gale OneFile) – 71 results
- CINAHL (EBSCOhost, 1981 - onward) – 82 results
- Cochrane CENTRAL (Ovid, July 2022) – 11 results
- Embase (Ovid, 1947 to 2022 July 26) – 230 results
- Érudit – 4 result
- Global Health (EBSCOhost, 1973 - onward) – 106 results
- LitCovid – 73 results
- MEDLINE (Ovid, 1946 to July 26, 2022) – 229 results
- Web of Science (Clarivate; Science Citation Index (1900-present), Social Sciences Citation Index (1900-present), Arts & Humanities Citation Index (1975-present), Emerging Sources Citation Index (2005-present)) – 124 results

The following strategies were used for each database.

APA PsycInfo (Ovid, 1806 to July Week 4 2022)

1. exp coronavirus/
2. coronavirus*.ti,ab.
3. (covid or covid19).ti,ab.

4. (2019-nCoV or 2019nCoV or nCoV2019 or nCov-2019 or SARS-CoV-2 or HCoV-19).ti,ab.
5. or/1-4
6. exp health attitudes/
7. treatment refusal/
8. ((vaccin* or immunis* or immuniz*) adj3 hesit*).ti,ab.
9. ((vaccin* or immunis* or immuniz*) adj3 (doubt* or uncertain* or indecis* or sceptic* or reluctan* or reservation* or ambivalan* or unwilling* or concern* or oppos* or distrust* or mistrust* or dropout* or criticism or fear* or refus* or reject* or rumo* or controvers* or misconc* or misinform* or object* or dilemma* or exempt* or declin* or delay*)).ti,ab.
10. ((vaccin* or immunis* or immuniz*) adj3 (accept* or confiden* or willing* or uptake* or decid* or decision* or adher* or comply or compliance or aware* or trust* or choice* or choos* or mandat* or compulsory or consen*)).ti,ab.
11. ((vaccin* or immunis* or immuniz*) adj3 (anxiet* OR anxious* or attitude* or belief* or perception or perceiv* or intent* or opinion* or knowledg* or prejudic* or behavio*)).ti,ab.
12. (antivaccin* or "anti-vaccin*" or "non-vaccin*" or nonvaccinat* or antivax or "anti-vax*").ti,ab.
13. or/6-12
14. (canad* OR "british columbia*" OR alberta* OR saskatchewan* OR manitoba* OR ontari* OR quebe* OR "new brunswick*" OR "nova scotia*" OR "prince edward island*" OR newfoundland* OR labrador* OR nunavut* OR nwt OR "northwest territor*" OR yukon*).mp
15. 5 and 13 and 14
16. limit 15 to yr="2020 -Current"

Cairn

1. (hésit* OR refus*) AND (vaccin* OR immunis*) AND (covid OR coronavirus)
   Search limited to the title and abstract fields

Canadian Business & Current Affairs (ProQuest, 1933 - current)

1. MAINSUBJECT.EXACT("Severe acute respiratory syndrome coronavirus 2") OR MAINSUBJECT.EXACT("Coronaviruses") OR MAINSUBJECT.EXACT("COVID-19")
2. MAINSUBJECT.EXACT("COVID-19 vaccines")
3. TI,AB(coronavirus*)
4. TI,AB(covid or covid19)
5. TI,AB(2019-nCoV or 2019nCoV or nCoV2019 or nCov-2019 or SARS-CoV-2 or HCoV-19)
6. 1 OR 2 OR 3 OR 4 OR 5
7. MAINSUBJECT.EXACT("Health behavior") OR MAINSUBJECT.EXACT("Treatment refusal")
8. TI,AB((vaccin* or immunis* or immuniz*) N/3 (hesit* OR doubt* or uncertain* or indecis* or sceptic* or reluctan* or reservation* or ambivalan* or unwilling* or concern* or oppos* or distrust* or mistrust* or dropout* or criticism or fear* or refus* or reject* or rumo* or controvers* or misconc* or misinform* or object* or dilemma* or exempt* or declin* or delay* OR accept* or confiden* or willing* or uptake* or decid* or decision* or adher* or comply or compliance))
9. TI,AB(((vaccin* or immunis* or immuniz*) N/3 (aware* or trust* or choice* or choos* or mandat* or compulsory or consen* OR anxiet* OR anxious* or attitude* or belief* or perception or perceiv* or intent* or opinion* or knowledg* or prejudic* or behavio*)) OR antivaccin* or "anti-vaccin*" or "non-vaccin*" or nonvaccinat* or antivax or "anti-vax*")

10. 6 AND 7
11. 6 AND 8
12. 6 AND 9
13. Due to limitations with the ProQuest interface, results from lines 10, 11 and 12 were selected and exported

    From results page, lines 10, 11 and 12 were limited to "Scholarly Journals" as Source Type and limited to 2020 to current as Publication date

Canadian Periodicals Index (Gale OneFile)

1. coronavirus* or covid or covid19 or 2019-nCoV or 2019nCoV or nCoV2019 or nCov-2019 or SARS-CoV-2 or HCoV-19
2. vaccin* or immunis* or immuniz*
3. hesit* OR doubt* or uncertain* or indecis* or sceptic* or reluctan* or reservation* or ambivalan* or unwilling* or concern* or oppos* or distrust* or mistrust* or dropout* or criticism or fear*
4. refus* or reject* or rumo* or controvers* or misconc* or misinform* or object* or dilemma* or exempt* or declin* or delay* OR accept* or confiden* or willing* or uptake* or decid* or decision* or adher* or comply or compliance
5. aware* or trust* or choice* or choos* or mandat* or compulsory or consen* OR anxiet* OR anxious* or attitude* or belief* or perception or perceiv* or intent* or opinion* or knowledg* or prejudic* or behavio*
6. antivaccin* or "anti-vaccin*" or "non-vaccin*" or nonvaccinat* or antivax or "anti-vax*"
7. canad* OR "british columbia*" OR alberta* OR saskatchewan* OR manitoba* OR ontari* OR quebe* OR "new brunswick*" OR "nova scotia*" OR "prince edward island*" OR newfoundland* OR labrador* OR nunavut* OR nwt OR "northwest territor*" OR yukon*

8. 1 AND 2 AND 3 AND 7
9. 1 AND 2 AND 4 AND 7
10. 1 AND 2 AND 5 AND 7
11. 1 AND 2 AND 6 AND 7
12. Due to limitations with the Gale OneFile interface, results from lines 8, 9, 10 and 11 were selected and exported

    From results page, lines 8, 9, 10 and 11 were limited to "Academic Journals" as Source Type and limited to 2020 to current as Publication date

CINAHL (EBSCOhost, 1981 current)

1. (MH "Coronavirus") OR (MH "Coronavirus Infections")
2. (MH "SARS-CoV-2") OR (MH "COVID-19 Pandemic") OR (MH "COVID-19")
3. (MH "COVID-19 Vaccines")
4. TI(coronavirus*) OR AB(coronavirus*)
5. TI(covid or covid19) OR AB(covid or covid19)
6. TI("2019-nCoV" or 2019nCoV or nCoV2019 or "nCov-2019" or "SARS-CoV-2" or "HCoV-19") OR AB("2019-nCoV" or 2019nCoV or nCoV2019 or "nCov-2019" or "SARS-CoV-2" or "HCoV-19")
7. S1 OR S2 OR S3 OR S4 OR S5 OR S6
8. (MH "Vaccination Coverage")
9. (MH "Health Behavior+")
10. (MH "Anti-Vaccination Movement")
11. TI((vaccin* or immunis* or immuniz*) N3 hesit*) OR AB((vaccin* or immunis* or immuniz*) N3 hesit*)
12. TI((vaccin* or immunis* or immuniz*) N3 (doubt* or uncertain* or indecis* or sceptic* or reluctan* or reservation* or ambivalan* or unwilling* or concern* or oppos* or distrust* or mistrust* or dropout* or criticism or fear* or refus* or

reject* or rumo* or controvers* or misconc* or misinform* or object* or dilemma* or exempt* or declin* or delay*)) OR AB((vaccin* or immunis* or immuniz*) N3 (doubt* or uncertain* or indecis* or sceptic* or reluctan* or reservation* or ambivalan* or unwilling* or concern* or oppos* or distrust* or mistrust* or dropout* or criticism or fear* or refus* or reject* or rumo* or controvers* or misconc* or misinform* or object* or dilemma* or exempt* or declin* or delay*))

13. TI((vaccin* or immunis* or immuniz*) N3 (accept* or confiden* or willing* or uptake* or decid* or decision* or adher* or comply or compliance or aware* or trust* or choice* or choos* or mandat* or compulsory or consen*)) OR AB((vaccin* or immunis* or immuniz*) N3 (accept* or confiden* or willing* or uptake* or decid* or decision* or adher* or comply or compliance or aware* or trust* or choice* or choos* or mandat* or compulsory or consen*))
14. TI((vaccin* or immunis* or immuniz*) N3 (anxiet* OR anxious* or attitude* or belief* or perception or perceiv* or intent* or opinion* or knowledg* or prejudic* or behavio*)) OR AB((vaccin* or immunis* or immuniz*) N3 (anxiet* OR anxious* or attitude* or belief* or perception or perceiv* or intent* or opinion* or knowledg* or prejudic* or behavio*))
15. TI(antivaccin* or "anti-vaccin*" or "non-vaccin*" or nonvaccinat* or antivax or "anti-vax*") OR AB(antivaccin* or "anti-vaccin*" or "non-vaccin*" or nonvaccinat* or antivax or "anti-vax*")
16. S8 OR S9 OR S10 OR S11 OR S12 OR S13 OR S14 OR S15
17. (MH "Canada+")
18. (canad* OR "british columbia*" OR alberta* OR saskatchewan* OR manitoba* OR ontari* OR quebe* OR "new brunswick*" OR "nova scotia*" OR "prince edward island*" OR newfoundland* OR labrador* OR nunavut* OR nwt OR "northwest territor*" OR yukon*)
19. S17 OR S18

20. S7 AND S16 AND S19
21. Used database limit from results screen to limit to 2020 onward.

Cochrane CENTRAL (Ovid)

1. exp coronavirus/
2. coronavirus*.ti,ab.
3. (covid or covid19).ti,ab.
4. (2019-nCoV or 2019nCoV or nCoV2019 or nCov-2019 or SARS-CoV-2 or HCoV-19).ti,ab.
5. or/1-4
6. exp attitude to health/
7. ((vaccin* or immunis* or immuniz*) adj3 hesit*).ti,ab.
8. ((vaccin* or immunis* or immuniz*) adj3 (doubt* or uncertain* or indecis* or sceptic* or reluctan* or reservation* or ambivalan* or unwilling* or concern* or oppos* or distrust* or mistrust* or dropout* or criticism or fear* or refus* or reject* or rumo* or controvers* or misconc* or misinform* or object* or dilemma* or exempt* or declin* or delay*)).ti,ab.
9. ((vaccin* or immunis* or immuniz*) adj3 (accept* or confiden* or willing* or uptake* or decid* or decision* or adher* or comply or compliance or aware* or trust* or choice* or choos* or mandat* or compulsory or consen*)).ti,ab.
10. ((vaccin* or immunis* or immuniz*) adj3 (anxiet* OR anxious* or attitude* or belief* or perception or perceiv* or intent* or opinion* or knowledg* or prejudic* or behavio*)).ti,ab.
11. (antivaccin* or "anti-vaccin*" or "non-vaccin*" or nonvaccinat* or antivax or "anti-vax*").ti,ab.
12. or/6-11
13. exp canada/

14. (canad* OR "british columbia*" OR alberta* OR saskatchewan* OR manitoba* OR ontari* OR quebe* OR "new brunswick*" OR "nova scotia*" OR "prince edward island*" OR newfoundland* OR labrador* OR nunavut* OR nwt OR "northwest territor*" OR yukon*).mp
15. or/13-14
16. 5 and 12 and 15
17. limit 16 to yr="2020 -Current"

Embase (Ovid, 1947 to 2022 July 26)

1. coronavirus infection/
2. exp coronavirus disease 2019/
3. coronavirus*.ti,ab
4. (covid or covid19).ti,ab
5. (2019-nCoV or 2019nCoV or nCoV2019 or nCov-2019 or SARS-CoV-2 or HCoV-19).ti,ab
6. or/1-5
7. vaccination hesitancy/
8. vaccination coverage/
9. exp treatment refusal/
10. attitude to health/
11. anti-vaccination movement/
12. ((vaccin* or immunis* or immuniz*) adj3 hesit*).ti,ab.
13. ((vaccin* or immunis* or immuniz*) adj3 (doubt* or uncertain* or indecis* or sceptic* or reluctan* or reservation* or ambivalan* or unwilling* or concern* or oppos* or distrust* or mistrust* or dropout* or criticism or fear* or refus* or reject* or rumo* or controvers* or misconc* or misinform* or object* or dilemma* or exempt* or declin* or delay*)).ti,ab.

14. ((vaccin* or immunis* or immuniz*) adj3 (accept* or confiden* or willing* or uptake* or decid* or decision* or adher* or comply or compliance or aware* or trust* or choice* or choos* or mandat* or compulsory or consen*)).ti,ab.
15. ((vaccin* or immunis* or immuniz*) adj3 (anxiet* OR anxious* or attitude* or belief* or perception or perceiv* or intent* or opinion* or knowledg* or prejudic* or behavio*)).ti,ab.
16. (antivaccin* or "anti-vaccin*" or "non-vaccin*" or nonvaccinat* or antivax or "anti-vax*").ti,ab.
17. or/7-16
18. exp canada/
19. (canad* OR "british columbia*" OR alberta* OR saskatchewan* OR manitoba* OR ontari* OR quebe* OR "new brunswick*" OR "nova scotia*" OR "prince edward island*" OR newfoundland* OR labrador* OR nunavut* OR nwt OR "northwest territor*" OR yukon*).mp
20. or/18-19
21. 6 and 17 and 20
22. limit 21 to yr="2020 -Current"

Érudit

1. (hésit* OR refus*) AND (vaccin* OR immunis*) AND (covid OR coronavirus)
   Search limited to the title, abstract and keyword fields

Global Health (EBSCOhost, 1973 - onward)

1. (DE "human coronaviruses" OR DE "Human coronavirus 229E" OR DE "Human coronavirus OC43" OR DE "Human enteric coronavirus")
2. (DE "severe acute respiratory syndrome" OR DE "Severe acute respiratory syndrome coronavirus")

3. TI(coronavirus*) OR AB(coronavirus*)
4. TI(covid or covid19) OR AB(covid or covid19)
5. TI("2019-nCoV" or 2019nCoV or nCoV2019 or "nCov-2019" or "SARS-CoV-2" or "HCoV-19") OR AB("2019-nCoV" or 2019nCoV or nCoV2019 or "nCov-2019" or "SARS-CoV-2" or "HCoV-19")
6. S1 OR S2 OR S3 OR S4 OR S5
7. TI((vaccin* or immunis* or immuniz*) N3 hesit*) OR AB((vaccin* or immunis* or immuniz*) N3 hesit*)
8. TI((vaccin* or immunis* or immuniz*) N3 (doubt* or uncertain* or indecis* or sceptic* or reluctan* or reservation* or ambivalan* or unwilling* or concern* or oppos* or distrust* or mistrust* or dropout* or criticism or fear* or refus* or reject* or rumo* or controvers* or misconc* or misinform* or object* or dilemma* or exempt* or declin* or delay*)) OR AB((vaccin* or immunis* or immuniz*) N3 (doubt* or uncertain* or indecis* or sceptic* or reluctan* or reservation* or ambivalan* or unwilling* or concern* or oppos* or distrust* or mistrust* or dropout* or criticism or fear* or refus* or reject* or rumo* or controvers* or misconc* or misinform* or object* or dilemma* or exempt* or declin* or delay*))
9. TI((vaccin* or immunis* or immuniz*) N3 (accept* or confiden* or willing* or uptake* or decid* or decision* or adher* or comply or compliance or aware* or trust* or choice* or choos* or mandat* or compulsory or consen*)) OR AB((vaccin* or immunis* or immuniz*) N3 (accept* or confiden* or willing* or uptake* or decid* or decision* or adher* or comply or compliance or aware* or trust* or choice* or choos* or mandat* or compulsory or consen*))
10. TI((vaccin* or immunis* or immuniz*) N3 (anxiet* OR anxious* or attitude* or belief* or perception or perceiv* or intent* or opinion* or knowledg* or prejudic* or behavio*)) OR AB((vaccin* or immunis* or immuniz*) N3 (anxiet* OR

anxious* or attitude* or belief* or perception or perceiv* or intent* or opinion* or knowledg* or prejudic* or behavio*))

11. TI(antivaccin* or "anti-vaccin*" or "non-vaccin*" or nonvaccinat* or antivax or "anti-vax*") OR AB(antivaccin* or "anti-vaccin*" or "non-vaccin*" or nonvaccinat* or antivax or "anti-vax*")
12. S7 OR S8 OR S9 OR S10 OR S11
13. DE "Canada" OR DE "British Columbia" OR DE "Manitoba" OR DE "New Brunswick" OR DE "Newfoundland and Labrador" OR DE "Northwest Territories" OR DE "Nova Scotia" OR DE "Nunavut" OR DE "Ontario" OR DE "Alberta" OR DE "Prince Edward Island" OR DE "Quebec" OR DE "Reindeer Lake" OR DE "Saskatchewan" OR DE "Yukon Territory"
14. (canad* OR "british columbia*" OR alberta* OR saskatchewan* OR manitoba* OR ontari* OR quebe* OR "new brunswick*" OR "nova scotia*" OR "prince edward island*" OR newfoundland* OR labrador* OR nunavut* OR nwt OR "northwest territor*" OR yukon*)
15. S13 OR S14
16. S6 AND S12 AND S15
17. Used database limit from results screen to limit to 2020 onward.

LitCovid

1. (canada OR canadian OR "british columbia" OR alberta OR saskatchewan OR manitoba OR ontario OR quebec OR "new brunswick" OR "nova scotia" OR "prince edward island" OR newfoundland OR labrador OR nunavut OR nwt OR "northwest territories" OR yukon) AND (hesitancy OR hesitant OR refusal OR "anti-vaccination" OR antivaccination OR antivax OR "anti-vax")

MEDLINE (Ovid, 1946 to July 26, 2022)

1. exp coronavirus/
2. covid-19/
3. covid-19 vaccines/
4. coronavirus*.ti,ab,kf.
5. (covid or covid19).ti,ab,kf.
6. (2019-nCoV or 2019nCoV or nCoV2019 or nCov-2019 or SARS-CoV-2 or HCoV-19).ti,ab,kf.
7. or/1-6
8. vaccination hesitancy/
9. vaccination refusal/ or vaccination coverage/
10. exp attitude to health/
11. anti-vaccination movement/
12. ((vaccin* or immunis* or immuniz*) adj3 hesit*).ti,ab,kf.
13. ((vaccin* or immunis* or immuniz*) adj3 (doubt* or uncertain* or indecis* or sceptic* or reluctan* or reservation* or ambivalan* or unwilling* or concern* or oppos* or distrust* or mistrust* or dropout* or criticism or fear* or refus* or reject* or rumo* or controvers* or misconc* or misinform* or object* or dilemma* or exempt* or declin* or delay*)).ti,ab,kf.
14. ((vaccin* or immunis* or immuniz*) adj3 (accept* or confiden* or willing* or uptake* or decid* or decision* or adher* or comply or compliance or aware* or trust* or choice* or choos* or mandat* or compulsory or consen*)).ti,ab,kf.
15. ((vaccin* or immunis* or immuniz*) adj3 (anxiet* OR anxious* or attitude* or belief* or perception or perceiv* or intent* or opinion* or knowledg* or prejudic* or behavio*)).ti,ab,kf.
16. (antivaccin* or "anti-vaccin*" or "non-vaccin*" or nonvaccinat* or antivax or "anti-vax*").ti,ab,kf.
17. or/8-16

18. exp canada/
19. (canad* OR "british columbia*" OR alberta* OR saskatchewan* OR manitoba* OR ontari* OR quebe* OR "new brunswick*" OR "nova scotia*" OR "prince edward island*" OR newfoundland* OR labrador* OR nunavut* OR nwt OR "northwest territor*" OR yukon*).mp
20. or/18-19
21. 7 and 17 and 20
22. limit 21 to yr="2020 -Current"

Web of Science (SCI, SSCI, AHCI, ESCI, 1900 - onward)

1. TS=(coronavirus*)
2. TS=(covid or covid19)
3. TS=(2019-nCoV or 2019nCoV or nCoV2019 or nCov-2019 or SARS-CoV-2 or HCoV-19)
4. #1 or #2 or #3
5. TS=((vaccin* or immunis* or immuniz*) near/3 hesit*)
6. TS=((vaccin* or immunis* or immuniz*) near/3 (doubt* or uncertain* or indecis* or sceptic* or reluctan* or reservation* or ambivalan* or unwilling* or concern* or oppos* or distrust* or mistrust* or dropout* or criticism or fear* or refus* or reject* or rumo* or controvers* or misconc* or misinform* or object* or dilemma* or exempt* or declin* or delay*))
7. TS=((vaccin* or immunis* or immuniz*) near/3 (accept* or confiden* or willing* or uptake* or decid* or decision* or adher* or comply or compliance or aware* or trust* or choice* or choos* or mandat* or compulsory or consen*))
8. TS=((vaccin* or immunis* or immuniz*) near/3 (anxiet* OR anxious* or attitude* or belief* or perception or perceiv* or intent* or opinion* or knowledg* or prejudic* or behavio*))

9. TS=(antivaccin* or "anti-vaccin*" or "non-vaccin*" or nonvaccinat* or antivax or "anti-vax*")
10. #5 OR #6 OR #7 OR #8 OR #9
11. TS=(canad* OR "british columbia*" OR alberta* OR saskatchewan* OR manitoba* OR ontari* OR quebe* OR "new brunswick*" OR "nova scotia*" OR "prince edward island*" OR newfoundland* OR labrador* OR nunavut* OR nwt OR "northwest territor*" OR yukon*)
12. #4 AND #10 AND #11
13. Used “Add Date Range” option when combining concepts to limit to 2020-2022 (Publication Date)
